# Supplementary material for: Chromothripsis during telomere crisis is independent of NHEJ, and consistent with a replicative origin
Source: Genome Res. 2019 May;29(5):737–49. doi: 10.1101/gr.240705.118 (PMC6499312; doi:10.1101/gr.240705.118)
Supplement: Supplemental Material [file supp_gr.240705.118_Supplemental_file_1.zip › contigs/annotated_contigs/DB78/contig.2.DB78_length_657_mean_cov_8.90410958904.docx]

**DB78_length_657_mean_cov_8.90410958904**

```
TTGTTGACAAAGCCAATGAATTATGTTATTCTTTCATTCAGTAAATATTTAATGCATAACTTTTATCTACAACGTGCTGTGGGGTGAAC
 >chr7:117732556-117732948 + E=4e-215
TGAAATTAAATGAAATTTATTTACTTAATAAATGTGTGATACAGTAAGAGAATAAGAGATGTGCAAACTCCTATAGTAGTAAGTTTACA

AGAAGTATTGGTGGTGAGCCAGAGATGTAGCGGAGAAGGTGGCATTTCACAGATCCTTAAAGAAATGATAAAATATGTAGATGGAGAAG

AAACTGGTGATGGGGATGGGATGGGGTGCTTAGTCAAAGCAGAAAAAAACTTCATAAGCAATGTTAGAGAGATCGTAAGAATCAGGTAC

TTATGGGGAGTAAGGAATATTTCCACTAAGGATG|AG|ACATAATTGTTCCCAATTACACTGTTTATCTCTGGAGTTTTGAGCATCTAT
                                   >chr7:117419406-117419673 + E=7e-149
ACTTCTAAAGGTTTTGTTCAGCTCATCAGCAACTAGTTGGAGAGAATGGATTTCTGTGTTCTCTAACTTAGGATGTGACTAATTTTTAT

GCTGTACTACTTATTTTTCATTCAGCCCTGTGATACAAAATTAATTCATTTTTCACTATAGAATTTTATACTGAATGTGTGTAACACCA

CAGATTTCTCAAGTAATATATATAAATAACATGAAA
```
